# Re: Reflections on the COVID-19 Pandemic

**DOI:** 10.1590/S1677-5538.IBJU.2020.0306

**Published:** 2020-04-23

**Authors:** Bertolo Riccardo, Chiara Cipriani, Matteo Vittori, Pierluigi Bove

**Affiliations:** 1 Department of Urology San Carlo di Nancy Hospital Rome Italy Department of Urology, San Carlo di Nancy Hospital, Rome, Italy;; 2 Department of Surgery Urology Unit Tor Vergata University of Rome Rome Italy Department of Surgery, Urology Unit, Tor Vergata University of Rome, Rome, Italy

To the editor,

We all know that Coronavirus disease 2019 (COVID-19) has been declared a pandemic on March 11th, 2020. Such dramatic scenario deeply impacted on the healthcare systems worldwide.

If telemedicine allows for the remote provision of healthcare by means of electronic communication tools in case of medical conditions, surgical indications could be not deferrable ( 1 , 2 ).

Major surgical societies have been prompted in publishing position papers and guidelines for best surgical practice. Among these, the European Association of Urology (EAU) Robotic Urology Section (ERUS) recently published its Guidelines on dealing with robotic surgery in the COVID-19 era ( 3 ).

Such guidelines include behavioral good clinical practice rules aimed to maximize the safety and the protection against COVID-19 for both patients and healthcare professionals involved in the robotic surgical activity. We followed the principles included in the ERUS guidelines either for pure-laparoscopic or robot-assisted procedures performed at our Institution since the beginning of the COVID-19 crisis. All patients with indication to surgery received preoperative health screening, with none of them reporting symptoms suggestive for COVID-19. Procedures were performed in a dedicated operative room. All the necessary protection tools and general recommendations to reduce the transmission of the disease were adequately followed ( 3 ). Selection of indications was considered in order to minimize the number of medical personnel involved and the expenditure of medical equipment. As such, only cystectomies, prostatectomies for high risk disease and renal surgeries for large renal masses were performed. All elective surgeries that could be delayed without any risk for the patient were postponed. Listed laparoscopic surgeries were performed at the lowest intra-abdominal pressure possible (8-10 mmHg), by using an intelligent integrated flow system (AirSeal®, ConMed, Utica, NY), allowing for system-assisted desufflation of the pneumoperitoneum. The minimum number of operative room staff members was adopted. No external observers, including residents and/or fellows, were allowed. Standardized surgical techniques were performed by experienced surgeons, in order to reduce the operative time and the risk of complications.

At the end of a three-weeks period, the teams involved in the operative room setting (including surgeons, anesthetists, nurses, operative room housekeepers and patients’ porters) were screened with a COVID-19 IgM/IgG rapid test lateral flow immunoassay, nowadays validated for the rapid diagnosis of COVID-19 ( 4 ).

VivaDiagTM COVID-19 IgM/IgG was performed according to the manufacturer’s instruction ( 5 ). After 15 minutes about, the result was read. Overall, > 300 tests were performed at our Institution. We focused on the 85 professionals who were related with the operative room activities reported herein. None of them resulted positive for either active or previous infection.

To date, real-time polymerase chain reaction in respiratory samples is the gold standard method for diagnosing COVID-19 ( 6 ). Nevertheless, molecular tests are time consuming, requiring specialized operators, thus limiting widespread use in real-life.

This is why we adopted VivaDiagTM COVID-19 IgM/IgG test. Although sensitivity has been published to be low ( 4 , 5 ), specificity is around 92%.

At a price running to 10 euros per person screened, we believe it could represent a value-for-money passport for immunity of health-care professionals.
